# Ammonium 4-meth­oxy­benzene­sulfonate

**DOI:** 10.1107/S1600536812028103

**Published:** 2012-06-27

**Authors:** Sebastián Suarez, Fabio Doctorovich, Ricardo Baggio

**Affiliations:** a Departamento de Química Inorgánica, Analítica y Química, Física/INQUIMAE–CONICET, Facultad de Ciencias Exactas y Naturales, Universidad de Buenos Aires, Buenos Aires, Argentina; b Gerencia de Investigación y Aplicaciones, Centro Atómico Constituyentes, Comisión Nacional de Energía Atómica, Buenos Aires, Argentina

## Abstract

The mol­ecular structure of the title compound, NH_4_^+^·C_7_H_7_O_4_S^−^, is featureless [the methoxy C atom deviating 0.173 (6) Å from the phenyl mean plane] with inter­atomic distances and angles in the expected ranges. The main feature of inter­est is the packing mode. Hydro­philic (SO_3_ and NH_4_) and hydro­phobic (PhOCH_3_) parts in the structure segregate, the former inter­acting through a dense hydrogen-bonding scheme, leading to a well connected two-dimensional structure parallel to (100) and the latter hydro­phobic groups acting as spacers for an inter­planar separation of *c*/2 = 10.205 (2) Å. In spite of being aligned along [110], the benzene rings stack in a far from parallel fashion [*viz.* consecutive ring centers determine a broken line with a 164.72 (12)° zigzag angle], thus preventing any possible π–π inter­action.

## Related literature

For literature on the role of weak inter­actions in supra­molecular structures, see: Desiraju (2007 ▶). For related structures, see: Fewings *et al.* (2001 ▶); Wang *et al.* (2007 ▶). For the Cambridge Structural Database, see: Allen (2002 ▶). For the synthesis, see: Porcheddu *et al.* (2009 ▶). 
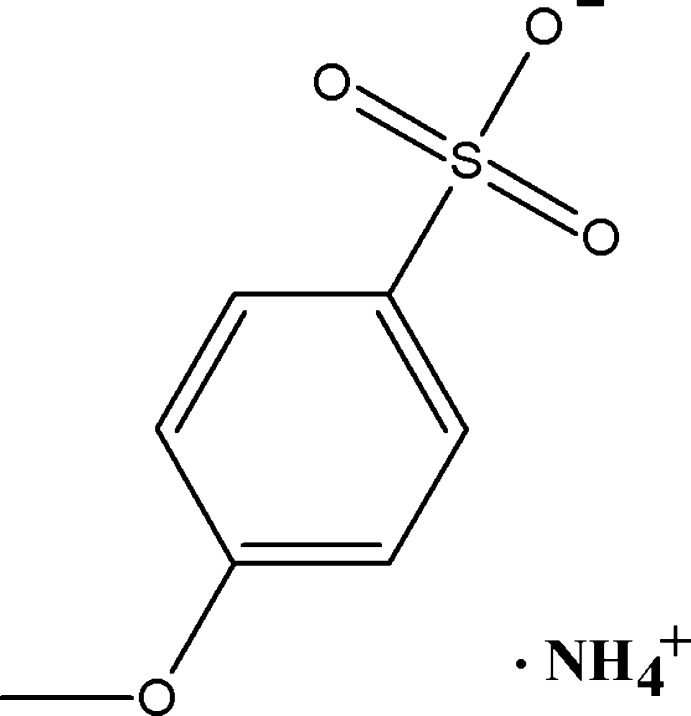


## Experimental

### 

#### Crystal data

NH_4_^+^·C_7_H_7_O_4_S^−^
*M* *_r_* = 205.23
Orthorhombic, 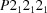

*a* = 6.2664 (12) Å
*b* = 7.1342 (12) Å
*c* = 20.410 (2) Å
*V* = 912.4 (2) Å^3^
*Z* = 4
Mo *K*α radiation
μ = 0.34 mm^−1^
*T* = 298 K
0.20 × 0.10 × 0.10 mm

#### Data collection

Oxford Diffraction Gemini CCD S Ultra diffractometer
Absorption correction: multi-scan (*CrysAlis PRO*; Oxford Diffraction, 2009 ▶) *T*_min_ = 0.958, *T*_max_ = 0.965
4265 measured reflections
1732 independent reflections
1548 reflections with *I* > 2σ(*I*)
*R*_int_ = 0.050

#### Refinement

*R*[*F* ^2^ > 2σ(*F* ^2^)] = 0.042
*wR*(*F* ^2^) = 0.119
*S* = 1.04
1732 reflections
135 parameters
21 restraints
H atoms treated by a mixture of independent and constrained refinement
Δρ_max_ = 0.47 e Å^−3^
Δρ_min_ = −0.36 e Å^−3^
Absolute structure: Flack (1983 ▶), 637 Friedel pairs
Flack parameter: −0.11 (14)

### 

Data collection: *CrysAlis PRO* (Oxford Diffraction, 2009 ▶); cell refinement: *CrysAlis PRO*; data reduction: *CrysAlis PRO*; program(s) used to solve structure: *SHELXS97* (Sheldrick, 2008 ▶); program(s) used to refine structure: *SHELXL97* (Sheldrick, 2008 ▶); molecular graphics: *SHELXTL* (Sheldrick, 2008 ▶); software used to prepare material for publication: *SHELXL97* and *PLATON* (Spek, 2009 ▶).

## Supplementary Material

Crystal structure: contains datablock(s) I, global. DOI: 10.1107/S1600536812028103/qm2074sup1.cif

Structure factors: contains datablock(s) I. DOI: 10.1107/S1600536812028103/qm2074Isup2.hkl

Supplementary material file. DOI: 10.1107/S1600536812028103/qm2074Isup3.cml

Additional supplementary materials:  crystallographic information; 3D view; checkCIF report

## Figures and Tables

**Table 1:** Hydrogen-bond geometry (Å, °)

*D*—H⋯*A*	*D*—H	H⋯*A*	*D*⋯*A*	*D*—H⋯*A*
N1—H1*N*⋯O1^i^	0.88 (2)	1.99 (2)	2.851 (3)	170 (3)
N1—H4*N*⋯O2^ii^	0.86 (2)	1.98 (2)	2.797 (3)	160 (3)
N1—H2*N*⋯O3^iii^	0.88 (2)	1.98 (2)	2.824 (3)	162 (3)
N1—H3*N*⋯O3	0.87 (2)	2.04 (2)	2.890 (3)	164 (3)

Symmetry codes: (i) 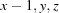; (ii) 
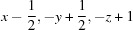
; (iii) 
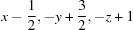
.

**Fig. 1.**
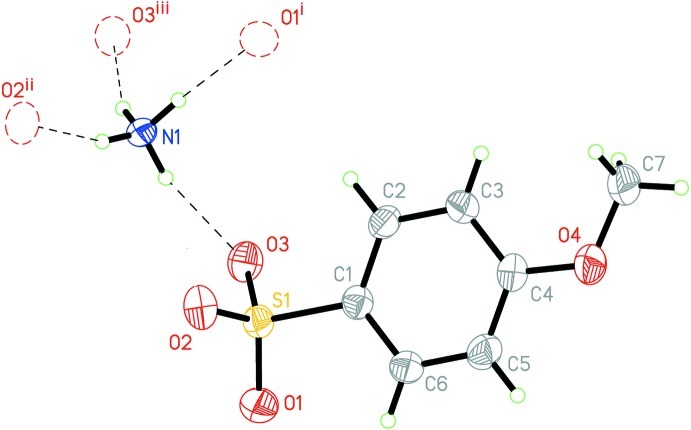
Ellipsoid plot of (I), drawn with displacement factors at a 50% probability level. Symmetry codes: (i) x - 1, y, z; (ii) x - 1/2, -y + 1/2, -z + 1; (iii) x - 1/2, -y + 3/2, -z + 1.

**Fig. 2.**
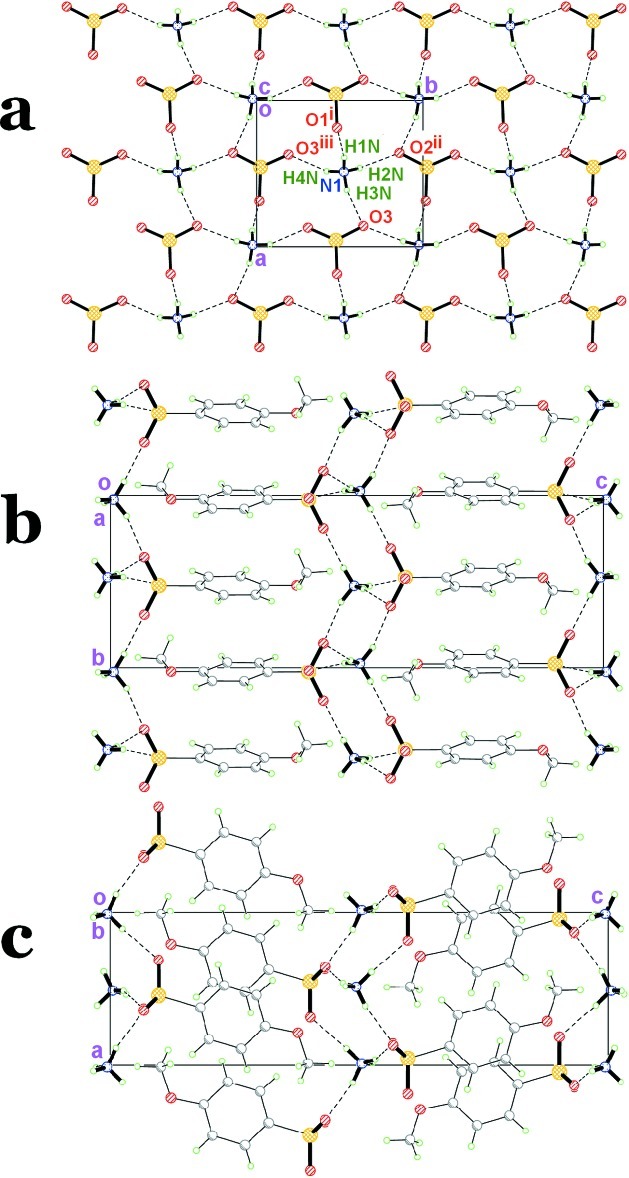
Packing views of (I). a) Projection paralell to (001) showing the hydrophilic part only and the H-bonding interactions taking place therein. Symmetry codes: as in Fig 1. b) A packing view with the whole structure, projected down [100]. Hydrophilic/hydrophobic parts (seen in projection) drawn in heavy/weak lining, respectively. c) Same as b) viewed along [010].

## supplementary crystallographic information

### Comment

The study of supramolecular systems determined by weak interactions such as hydrogen bonding, π-π stacking or dipole- dipole interactions have been, and currently are, active fields of structural research due to their implications in crystal engineering, self-assembly and, above all, biological systems (Desiraju, 2007). Derivatives of the benzenesulfonate anion are extremely suited to this end due to the possibility of π-interactions between arene rings, as well as hydrogen bonding between the sulphonate groups and any H donor eventualy available (Water, ammonium, *etc*). With this latter NH_4_ partner a number a structures of the sort have been published (among many others, ammonium *p*-toluenesulfonate, Fewings *et al.*, 2001, (II); ammonium 4-hydroxybenzenesulfonate, Wang *et al.*, 2007, (III), *etc*), the vast majority displaying, as expected, an extremely complex non-bonding interactions scheme. We present herein one further member in this family, ammonium 4-methoxybenzenesulfonate, C_7_H_7_O_4_S.H_4_N (I), which ended up being isotructural to (II) but different from (III), in spite of the very similar formulations.

The molecular structure in (I) (Fig 1) is featureless, with interatomic bond and angles in the expected ranges, and its main interest resides in the packing mode. Hydrophilic (SO_3_, NH_4_) and hydrophobic (PhOCH_3_) parts in the structure segregate, the former one interacting through a dense H-bonding scheme (Table 1) leading to a well connected two-dimensional structure, parallell to (100) (Fig 2a) and the latter hydrophobic groups acting as spacers (Figs 2 b, 2c), for an interplanar separation of C/2 = 10.205 (2) Å. In spite of the deceiving views in Figs 2 b/2c, Ph groups stack in a far from paralell fashion, defining dihedral angles of 37° and thus preventing any possible π–π interaction.

### Experimental

The title compound was obtained as a byproduct in the synthesis of *N*-hydroxy-4-methoxybenzenesulfonamide, following the procedure described in Porcheddu *et al.*, 2009. A few light yellow crystals were obtained after evaporating an acetonitrile solution.

### Refinement

All H atoms were found in a difference map, though treated differently in refinement: C—H atoms were idealized and allowed to ride, with displacement parameters taken as *U*_iso_(H) = *X*×*U*_eq_(C) [(*C*—*H*)_methyl_ = 0.96 A°, *X* = 1.5; (C—H)_arom_ = 0.93 A°, *X* = 1.2] (CH_3_ groups were also free to rotate as well). Ammonium H's were refined with restrained N—H = 0.85 (1) Å, H···H = 1.35 (2) Å distances and free isotropic displacement factors.

### Crystal data

**Table:**

NH_4_^+^·C_7_H_7_O_4_S^−^	*F*(000) = 432
*M**_r_* = 205.23	*D*_x_ = 1.494 Mg m^−^^3^
Orthorhombic, *P*2_1_2_1_2_1_	Mo *K*α radiation, λ = 0.71073 Å
Hall symbol: P 2ac 2ab	Cell parameters from 2823 reflections
*a* = 6.2664 (12) Å	θ = 2.1–25.9°
*b* = 7.1342 (12) Å	µ = 0.34 mm^−^^1^
*c* = 20.410 (2) Å	*T* = 298 K
*V* = 912.4 (2) Å^3^	Blocks, yellow
*Z* = 4	0.20 × 0.10 × 0.10 mm

### Data collection

**Table:**

Oxford Diffraction Gemini CCD S Ultra diffractometer	1548 reflections with *I* > 2σ(*I*)
Graphite monochromator	*R*_int_ = 0.050
ω scans, thick slices	θ_max_ = 26.2°, θ_min_ = 2.0°
Absorption correction: multi-scan (*CrysAlis PRO*; Oxford Diffraction, 2009)	*h* = −7→6
*T*_min_ = 0.958, *T*_max_ = 0.965	*k* = −8→8
4265 measured reflections	*l* = −20→25
1732 independent reflections	

### Refinement

**Table:**

Refinement on *F*^2^	Secondary atom site location: difference Fourier map
Least-squares matrix: full	Hydrogen site location: inferred from neighbouring sites
*R*[*F*^2^ > 2σ(*F*^2^)] = 0.042	H atoms treated by a mixture of independent and constrained refinement
*wR*(*F*^2^) = 0.119	*w* = 1/[σ^2^(*F*_o_^2^) + (0.0842*P*)^2^] where *P* = (*F*_o_^2^ + 2*F*_c_^2^)/3
*S* = 1.04	(Δ/σ)_max_ < 0.001
1732 reflections	Δρ_max_ = 0.47 e Å^−^^3^
135 parameters	Δρ_min_ = −0.36 e Å^−^^3^
21 restraints	Absolute structure: Flack (1983), 637 Friedel pairs
Primary atom site location: structure-invariant direct methods	Flack parameter: −0.11 (14)

### Special details

**Table:**

Geometry. All e.s.d.'s (except the e.s.d. in the dihedral angle between two l.s. planes) are estimated using the full covariance matrix. The cell e.s.d.'s are taken into account individually in the estimation of e.s.d.'s in distances, angles and torsion angles; correlations between e.s.d.'s in cell parameters are only used when they are defined by crystal symmetry. An approximate (isotropic) treatment of cell e.s.d.'s is used for estimating e.s.d.'s involving l.s. planes.

### Fractional atomic coordinates and isotropic or equivalent isotropic displacement parameters (Å^2^)

**Table:**

	*x*	*y*	*z*	*U*_iso_*/*U*_eq_	
S1	0.95549 (11)	0.47643 (10)	0.59811 (3)	0.0305 (2)	
O1	1.1864 (3)	0.4832 (4)	0.59781 (11)	0.0483 (6)	
O2	0.8694 (4)	0.3085 (3)	0.56840 (11)	0.0422 (6)	
O3	0.8578 (4)	0.6413 (3)	0.56824 (11)	0.0379 (6)	
O4	0.7122 (4)	0.4778 (4)	0.87726 (9)	0.0426 (6)	
C1	0.8765 (4)	0.4753 (4)	0.68155 (13)	0.0295 (6)	
C2	0.6702 (4)	0.5282 (5)	0.69849 (13)	0.0324 (6)	
H2	0.5734	0.5616	0.6660	0.039*	
C3	0.6087 (4)	0.5311 (5)	0.76355 (13)	0.0338 (6)	
H3	0.4713	0.5677	0.7751	0.041*	
C4	0.7550 (5)	0.4783 (4)	0.81175 (13)	0.0324 (6)	
C5	0.9583 (6)	0.4232 (4)	0.79423 (15)	0.0375 (7)	
H5	1.0544	0.3863	0.8265	0.045*	
C6	1.0207 (5)	0.4223 (4)	0.72898 (14)	0.0335 (6)	
H6	1.1583	0.3864	0.7174	0.040*	
C7	0.5125 (6)	0.5513 (6)	0.89746 (15)	0.0498 (8)	
H7A	0.5091	0.5598	0.9444	0.075*	
H7B	0.4001	0.4700	0.8828	0.075*	
H7C	0.4931	0.6737	0.8789	0.075*	
N1	0.4879 (3)	0.5244 (3)	0.49420 (10)	0.0264 (5)	
H1N	0.391 (3)	0.526 (4)	0.5251 (9)	0.034 (8)*	
H2N	0.463 (5)	0.619 (3)	0.4678 (12)	0.068 (13)*	
H3N	0.612 (3)	0.542 (5)	0.5125 (10)	0.039 (9)*	
H4N	0.484 (5)	0.421 (3)	0.4729 (13)	0.069 (14)*	

### Atomic displacement parameters (Å^2^)

**Table:**

	*U*^11^	*U*^22^	*U*^33^	*U*^12^	*U*^13^	*U*^23^
S1	0.0297 (3)	0.0358 (3)	0.0261 (3)	−0.0015 (3)	0.0015 (3)	−0.0013 (3)
O1	0.0310 (11)	0.0745 (17)	0.0394 (12)	0.0018 (12)	0.0032 (9)	−0.0021 (15)
O2	0.0533 (16)	0.0399 (12)	0.0336 (12)	−0.0030 (10)	0.0042 (12)	−0.0044 (10)
O3	0.0454 (13)	0.0370 (11)	0.0313 (12)	−0.0024 (10)	−0.0004 (11)	0.0042 (10)
O4	0.0491 (12)	0.0521 (13)	0.0265 (10)	0.0068 (12)	−0.0010 (9)	0.0003 (11)
C1	0.0305 (12)	0.0307 (13)	0.0273 (13)	−0.0035 (12)	0.0009 (11)	−0.0001 (12)
C2	0.0300 (13)	0.0393 (14)	0.0279 (13)	−0.0026 (13)	−0.0045 (11)	0.0008 (14)
C3	0.0286 (13)	0.0402 (15)	0.0327 (14)	0.0001 (12)	0.0023 (11)	−0.0037 (14)
C4	0.0390 (14)	0.0313 (13)	0.0268 (13)	−0.0036 (13)	−0.0006 (11)	−0.0015 (13)
C5	0.0416 (16)	0.0377 (15)	0.0331 (15)	0.0081 (14)	−0.0069 (14)	0.0024 (12)
C6	0.0331 (15)	0.0333 (13)	0.0342 (14)	0.0060 (12)	−0.0021 (12)	−0.0019 (11)
C7	0.0438 (17)	0.076 (2)	0.0292 (15)	−0.0005 (18)	0.0048 (14)	−0.0050 (17)
N1	0.0247 (10)	0.0303 (10)	0.0241 (10)	0.0038 (9)	−0.0026 (9)	0.0019 (10)

### Geometric parameters (Å, º)

**Table:**

S1—O2	1.447 (2)	C4—C5	1.380 (4)
S1—O1	1.448 (2)	C5—C6	1.388 (4)
S1—O3	1.459 (2)	C5—H5	0.9300
S1—C1	1.773 (3)	C6—H6	0.9300
O4—C4	1.364 (3)	C7—H7A	0.9600
O4—C7	1.418 (4)	C7—H7B	0.9600
C1—C6	1.377 (4)	C7—H7C	0.9600
C1—C2	1.390 (4)	N1—H1N	0.876 (15)
C2—C3	1.383 (4)	N1—H2N	0.877 (16)
C2—H2	0.9300	N1—H3N	0.873 (16)
C3—C4	1.396 (4)	N1—H4N	0.858 (16)
C3—H3	0.9300		
			
O2—S1—O1	113.48 (17)	C4—C5—C6	120.7 (3)
O2—S1—O3	109.62 (13)	C4—C5—H5	119.7
O1—S1—O3	112.99 (16)	C6—C5—H5	119.7
O2—S1—C1	107.15 (14)	C1—C6—C5	119.2 (3)
O1—S1—C1	106.45 (13)	C1—C6—H6	120.4
O3—S1—C1	106.71 (14)	C5—C6—H6	120.4
C4—O4—C7	117.2 (2)	O4—C7—H7A	109.5
C6—C1—C2	120.6 (3)	O4—C7—H7B	109.5
C6—C1—S1	119.6 (2)	H7A—C7—H7B	109.5
C2—C1—S1	119.8 (2)	O4—C7—H7C	109.5
C3—C2—C1	120.1 (3)	H7A—C7—H7C	109.5
C3—C2—H2	119.9	H7B—C7—H7C	109.5
C1—C2—H2	119.9	H1N—N1—H2N	108 (3)
C2—C3—C4	119.3 (3)	H1N—N1—H3N	108 (3)
C2—C3—H3	120.4	H2N—N1—H3N	108 (3)
C4—C3—H3	120.4	H1N—N1—H4N	111 (3)
O4—C4—C5	115.8 (3)	H2N—N1—H4N	110 (3)
O4—C4—C3	124.2 (3)	H3N—N1—H4N	111 (3)
C5—C4—C3	120.0 (3)		

### Hydrogen-bond geometry (Å, º)

**Table:**

*D*—H···*A*	*D*—H	H···*A*	*D*···*A*	*D*—H···*A*
N1—H1*N*···O1^i^	0.88 (2)	1.99 (2)	2.851 (3)	170 (3)
N1—H4*N*···O2^ii^	0.86 (2)	1.98 (2)	2.797 (3)	160 (3)
N1—H2*N*···O3^iii^	0.88 (2)	1.98 (2)	2.824 (3)	162 (3)
N1—H3*N*···O3	0.87 (2)	2.04 (2)	2.890 (3)	164 (3)

Symmetry codes: (i) *x*−1, *y*, *z*; (ii) *x*−1/2, −*y*+1/2, −*z*+1; (iii) *x*−1/2, −*y*+3/2, −*z*+1.

## References

[bb1] Allen, F. H. (2002). *Acta Cryst.* B**58**, 380–388. 10.1107/s0108768102003890 12037359

[bb2] Desiraju, G. R. P. (2007). *Angew. Chem. Int. Ed.* **46**, 8342–8356. 10.1002/anie.200700534 17902079

[bb3] Fewings, K. R., Junk, P. C., Georganopoulou, D., Prince, P. D. & Steed, J. W. (2001). *Polyhedron*, **20**, 643–649.

[bb4] Flack, H. D. (1983). *Acta Cryst.* A**39**, 876–881.

[bb5] Oxford Diffraction (2009). *CrysAlis PRO* Oxford Diffraction Ltd, Yarnton, Oxfordshire, England.

[bb6] Porcheddu, A., De Luca, L. & Giacomelli, G. (2009). *Synlett*, **13**, 2149–2153.

[bb7] Sheldrick, G. M. (2008). *Acta Cryst.* A**64**, 112–122. 10.1107/S0108767307043930 18156677

[bb8] Spek, A. L. (2009). *Acta Cryst.* D**65**, 148–155. 10.1107/S090744490804362X PMC2631630 19171970

[bb9] Wang, K.-W., Feng, W.-J., Li, H.-Y., Ma, L.-L. & Jin, Z.-M. (2007). *Acta Cryst.* E**63**, o3481.

